# Phenotypic and Transcriptomic Analysis of Two *Pinellia ternata* Varieties T2 line and T2Plus line

**DOI:** 10.1038/s41598-020-61512-2

**Published:** 2020-03-12

**Authors:** Jun Lu, Jian Ning Liu, Surendra Sarsaiya, Gregory Joseph Duns, Jing Han, Leilei Jin, Jishuang Chen

**Affiliations:** 1 College of Biotechnology and Pharmaceutical Engineering, Nanjing Tech University, Nanjing, 211800 Jiangsu China; 2 KeGene Science & Technology Co. Ltd., Nantianmen Middle Road, Tai’an, 271018 China; 3 Bioresource Institute for Healthy Utilization, Zunyi Medical University, Zunyi, 563000 Guizhou China; 4 AirChem Consulting and Research, London, Ontario N5X OE2 Canada

**Keywords:** Transcriptomics, Medical research

## Abstract

*Pinellia* (*Pinellia ternata* (Thunb.) Breit.), as important medicinal plant, has been used to treat various ailments for a long time. The sixteen ploid plant (2n = 16 * 13 = 208) *Pinellia* T2Plus line was obtained from an octoploid (2n = 8 * 13 = 104) T2 line by chromosome-doubling technique. Compared with T2 line, the content of various medicinal components (polysaccharide, guanosine, adenosine and ephedrine) was increased in T2Plus line. In this study, the transcriptome of T2 line and T2Plus line were characterized by RNA sequencing (RNA-seq) technology. Gene Ontology (GO) and Kyoto Encyclopedia of Genes and Genomes (KEGG) pathways enrichment analysis on differential expressed unigenes (DEGs) revealed that multiple metabolic pathway were enriched significantly, such as ‘Starch and sucrose metabolism’, ‘Purine metabolism’, ‘Photosynthesis’ and six transcription factors (MYB, WRKY, bHLH, lateral organ boundaries domain (LBD), homeodomain-zipper (HD-ZIP) and Ethylene-responsive factor (ERF)) play a key role in difference of transcriptome between T2 line and T2Plus line. These metabolic pathways and transcription factors may play an important role in the difference of medicinal components and epigenetic features between these two *Pinellia* cultivars. This conclusion provides a robust theoretical basis for the mechanism of the formation of medicinal ingredients in *Pinellia* cultivars.

## Introduction

*Pinellia* (*Pinellia ternata* (Thunb.) Breit.), mainly distributed in Eastern Asia, has been used to treat various ailments for a long time, such as vomiting, inflammation, cough, epilepsy, cervical cancer and traumatic injury, etc^1^. A wide range of biological activities in *Pinellia* was revealed by pharmacological studies and these activities include sedative and hypnotic^2^, anti-tumor^3^, anti-emetic^4^, anti-inflammatory and antitussive^5^, antibacterial and anti-inflammatory^6^. Due to the higher medicinal value and longer life cycle of *Pinellia*, overexploitation of the wild resources of this *Pinellia* led to the phenomenon of undersupply under-supply. Therefore, it is necessary to obtain high-yield *Pinellia* cultivar to sustain balance both the market and the abundance of this species.

Genomic polyploidization not only produce new phenotypic traits, but also stronger environmental/stress adaptation, thereby enabling diversification and speciation and driving the evolution and divergence in gene function and plant behavior^7^. The polyploid genome is affected the gene expression in response to stress and hormones, thus polyploids tend to exhibit greater tolerance in salt, heat and drought^8–12^,. In addition, the yield of polyploid plants is generally increased. For example, polyploid *sugar beet* is provided with the characteristics of nutrient growth, high root yield and high sugar content^13^. Artificially induced plant polyploids showing excellent traits have been widely used in agriculture, providing abundant germplasm resources for agricultural production^14–18^.

The apparent characteristics of plants are closely related to the expression of genes *in vivo*. After chromosome doubling, some genes will increase expression due to dose effects, some will attenuate expression due to gene silencing effects, and some will not change much. In order to better explore the relationship between chromosome doubling and epigenetics in *P. ternata*, it is necessary to conduct in-depth research at the omics level. Therefore, RNA-seq experiments were performed using total RNA isolated from octoploid *Pinellia* line T2 (2n = 8 * 13 = 104) and sixteen ploid *Pinellia* line T2Plus (2n = 16 * 13 = 208). In T2Plus line, many genes and transcription factors related to multiple metabolic pathways were expressed up-regulated significantly. Our results indicate that these up-regulated genes and transcription factors may be involved in the bio-synthesis of medicinal ingredients.

## Results

### Comparison of biological characteristics

**Figure 1:**
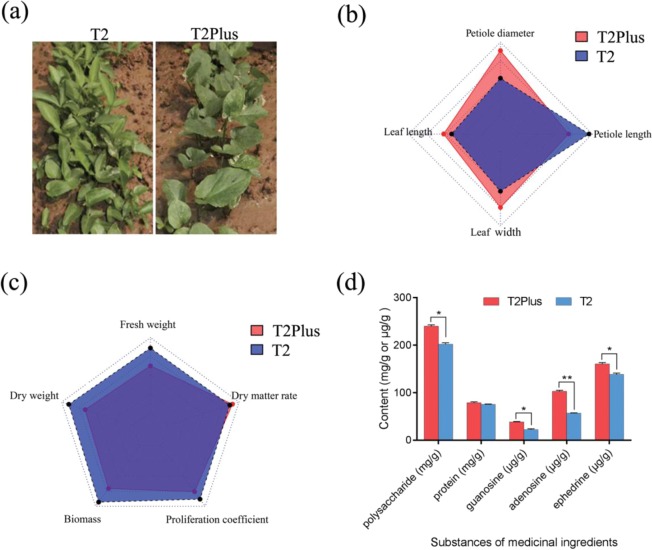
Analysis of biological characteristics of two *P. ternata* cultivars (T2 line and T2Plus line). (**a**) Field growth situations of two *P. ternata* lines. (**b**) Appearance morphology of *P. ternata* T2Plus line compared with T2 line. (**c**) Comparison of proliferation of *P. ternata* T2Plus line and T2 line. (**d**) Content of these substances (polysaccharide, protein, guanosine, adenosine, ephedrine) of tubers in *P. ternata* were measured (*p* ≤ 0.05; two-tailed Student’s t-test). Error bars represent standard error of the mean (n = 3).

To solve the problem of uneven quality of *Pinellia* plants, a superior individual T2 line was obtained by screening cultivar from different regions. Tissue culturing, a technique that enables plants to rapidly reproduce in a sterile environment to achieve detoxification, was performed to generate a high-yield individual cultivar line T2 without contamination viruses or bacteria. Based on the high-quality T2 seedlings, the sixteen ploid *Pinellia* T2Plus line was obtained by using a chromosome-doubling technique (colchicine-induced). The T2 line, as a single plant Shandong Heze peach leaf Banxia 2 (T2), is an octoploid line existing in nature (2n = 8 * 13 = 104), However, the T2Plus line, as a sixteen times body (2n = 16 * 13 = 208) that does not exist in nature, has high research value. To identify the differences between T2 line and T2Plus line, we analyzed the biological characteristics of these two cultivars. It was found that as the number of leaves increased, the tuber also increased rapidly, but the phenotype in the T2Plus line were significantly better than those in T2 line (Fig. 1a). At the leaf proliferative phase, compared with T2 line, the length of the petiole was shorter, the diameter of the petiole was larger and the ratio of leaf length to width was smaller in T2Plus line (Figs. 1b, S1). At the seedling stage, compared with T2 line, T2Plus line had a low proliferation coefficient and less biomass accumulation (Fig. 1c) showed that T2Plus line has a later germination period and slow growth.

*Pinellia* is an important Chinese herbal medicine, therefore it is necessary to investigate any changes of the medicinal ingredients between T2 line and T2Plus line. There are many substances as the main components of the medicinal ingredients. For example, polysaccharides are the main components of the anti-tumor effect in the tuber of *P. ternata*^19^. Lectin, as a kind of protein, plays an important role in anti-tumor, anti-growth and anti-aphid effects^20^. Alkaloids are an essential ingredient in anti-tumor and lipid-lowering applications, as well as for treatment of cough, vomiting and other aspects^21^. The nucleoside substances of *P. ternata* such as guanine nucleoside, adenine nucleoside and hypoxanthine nucleoside, as water-soluble components, are involved in DNA metabolism, energy metabolism and protein synthesis. They can also improve liver function, contribute to the recovery of damaged liver cell function, stimulate the generation of antibodies in the body, and improve the intestinal absorption of iron^22^. Therefore, the possible changes of these substances (polysaccharides, proteins, guanosine, adenosine, ephedrine) of tubers in *P. ternata* were measured. Compared with T2 line, the contents of polysaccharides, guanosine, adenosine and ephedrine in T2Plus line were significantly higher and the content of protein remained unchanged (Fig. 1d). These results suggest that the content of medicinal ingredients is increased after the doubling of the chromosome number in *P. ternata*.

### Quantification and quality assessment of transcriptome data of *P. ternata*

**Figure 2:**
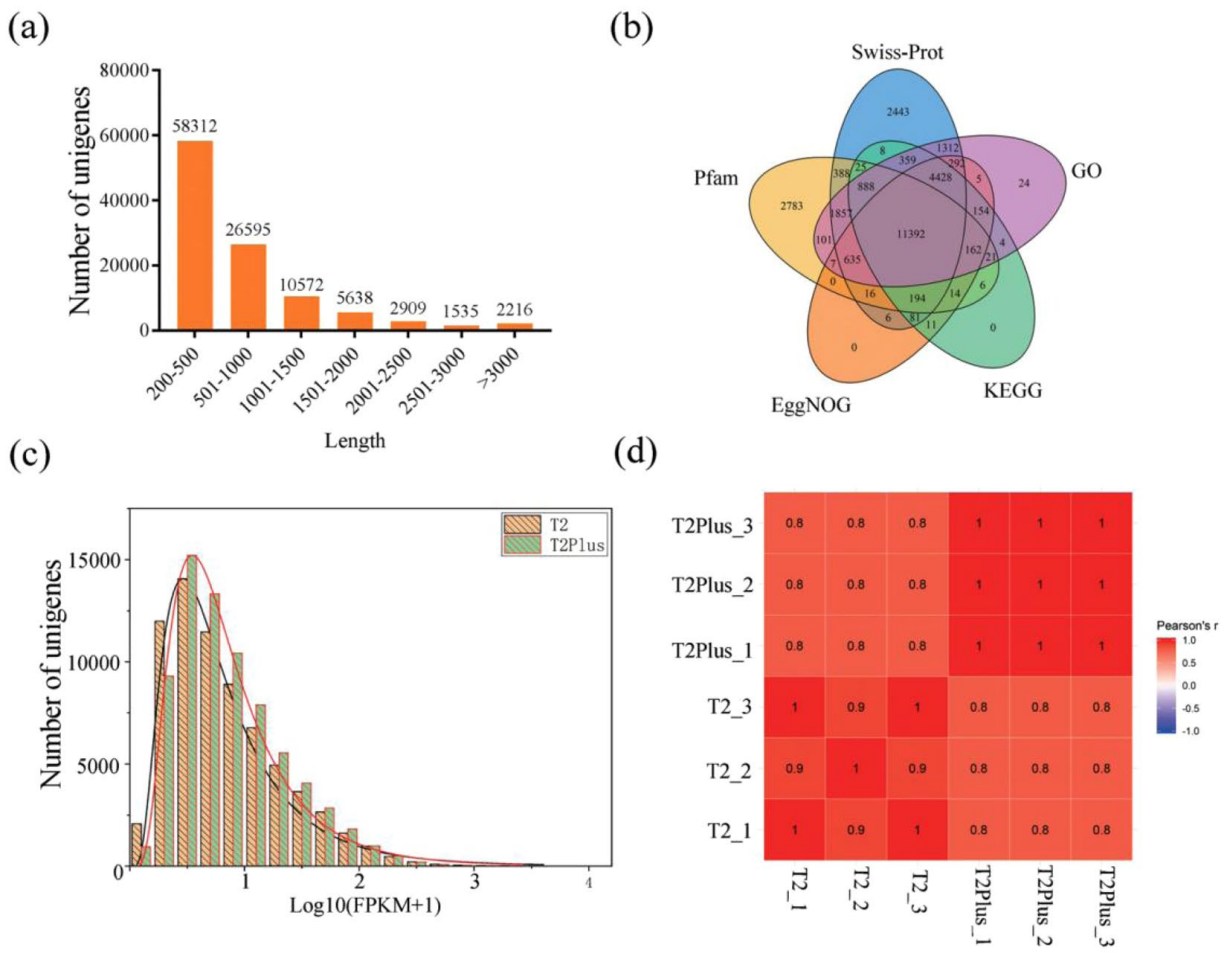
Analysis of the characteristics of *P. ternata* transcriptome. (**a**) Length distribution of assembled unigenes. (**b**) The number of unigenes annotated into five databases (Swiss-Prot, Pfam, EggNOG, KEGG, GO) is shown. (**c**) Distribution plot for average expressions of unigenes in T2 line and T2Plus line. (**d**) Heatmaps showing correlation between transcriptomes of three biological replicates of each cultivar. Pearson correlation coefficient among the replicates of Pinellia *Pinellia* are shown.

To investigate the transcriptome differences between T2 line and T2Plus line, we performed RNA-seq experiments using total RNA isolated from these two lines. These two cultivars were analyzed in three independent biological replicates (6 samples in total). Total RNA was extracted from the *Pinellia* seedlings grown for 30 days, and cDNA libraries were prepared and sequenced with Illumina HiSeq sequencing instrument. After removing adaptor sequences, ambiguous reads and low-quality reads from 293,790,298 raw reads, a total of 255,299,598 clean reads (87.01% of raw reads) were generated, of which the mean length was 150 bp (Table 1). A total of 116,434 transcripts and 107,777 unigenes were generated by *De novo* assembly using Trinity Software. In these unigenes, 58,312 unigenes (54.10%) were between 200 and 500 bp in length, 26,595 unigenes (24.68%) were between 500 and 1000 bp and 22,870 unigenes (21.22%) were longer than 1000 nt (Fig. 2a). The functional information of these unigenes was obtained from five public protein databases (Swiss-Prot, Protein family (Pfam), evolutionary genealogy of genes:Non-supervised Orthologous Groups (EggNOG), KEGG, GO) (E value < 10^−5^) by performing basic local alignment search tool nucleic acid (BLASTN) (Table S1). A total of 27,616 (25.62%) unigenes were annotated in at least one database and 11,392 (10.57%) unigenes were annotated in all five the databases (Fig. 2b).

**Table 1:** Output of the clean reads from transcriptome data of *P. ternata*.

Samples	Raw Reads	Clean Reads	Clean Base (Gb)	Reads Length	Validity (%)
T2-1	48476126	41934026	6.29	150	86.5
T2-2	70238152	60303568	9.05	150.07	85.86
T2-3	43469330	37532218	5.63	150	86.34
T2p-1	46956466	41336168	6.2	149.99	88.03
T2p-2	40078230	35364586	5.3	149.87	88.24
T2p-3	44571994	38829032	5.82	149.89	87.12
All samples	293790298	255299598	38.29	149.97	87.015

The uniquely mapped reads for each sample were processed by Expectation-Maximization (RSEM) to determine the normalized expression level of each unigene (Fig. S2; Table S2). The expression level of unigenes in T2Plus line was higher than that of T2 line (Fig. 2c). The Pearson correlation coefficient between the biological replicates of different samples varied from 0.94 to 0.98, indicating the high quality of the replicates (Fig. 2d).

### Identification and annotation of differentially expressed genes between T2 line and T2Plus line

**Figure 3:**
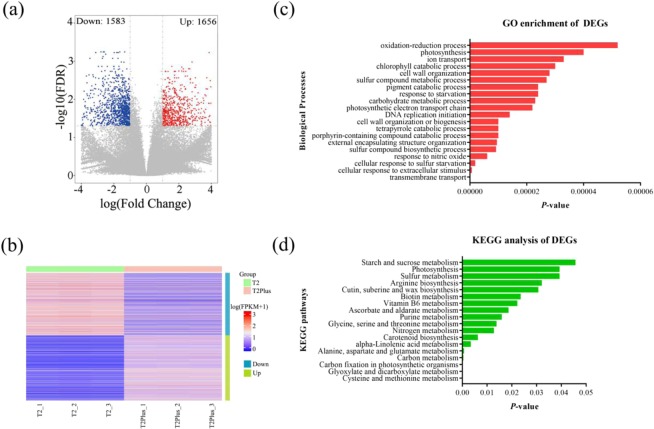
Identification and functional information of differential expression genes (DEGs) in T2Plus line as compared with T2 line. (**a**) volcano of differentially expressed unigenes in both groups. (**b**) The expression profile of all DEGs was shown by heatmap. (**c**) The top 20 GO terms (rank by *P*-value) of biological processes were listed by histogram. (**d**) Histogram shown KEGG pathways enriched significantly (*P* ≤ 0.05).

We identified gene sets showing significant differential expression between these two cultivars cultivar. In total, 3,239 differential expression genes (DEGs; false discovery rate (FDR) ≤ 0.05, |log2FC | ≥ 1) were generated, including 1,656 up-regulated DEGs and 1,583 down-regulated DEGs in T2Plus line (Fig. 3a,b; Table S3). The GO enrichment analysis of DEGs in T2Plus line was performed and the top 20 GO terms (ranked by *p*-value) of biological processes were listed (Fig. 3c; Table S4). Various cell-related terms, such as cell cycle, cell wall organization or bio-genesis, cell division and mitotic cell cycle, were significantly enriched in DEGs. In addition, GO terms, photosynthesis, photosynthetic electron transport chain and chlorophyll catabolic processes were also highly enriched in DEGs (Fig. 3c; Table S4).

To investigate the changes of metabolic pathways between T2 line and T2Pus line, all DEGs were subjected to Kyoto Encyclopedia of Genes and Genomes (KEGG) pathways analysis and the significant pathways (*P* ≤ 0.05) were shown (Fig. 3d; Table S4). A total of 18 metabolic pathways were enriched significantly, including ‘Carbon fixation in photosynthetic organisms’, ‘Purine metabolism’, ‘Photosynthesis’ and ‘Starch and sucrose metabolism’, etc (Fig. 3d), suggesting that these metabolic pathways may associate with development and the synthesis of medicinal ingredients in *Pinellia*.

### DEGs related to starch and sucrose metabolism and purine metabolism

**Figure 4:**
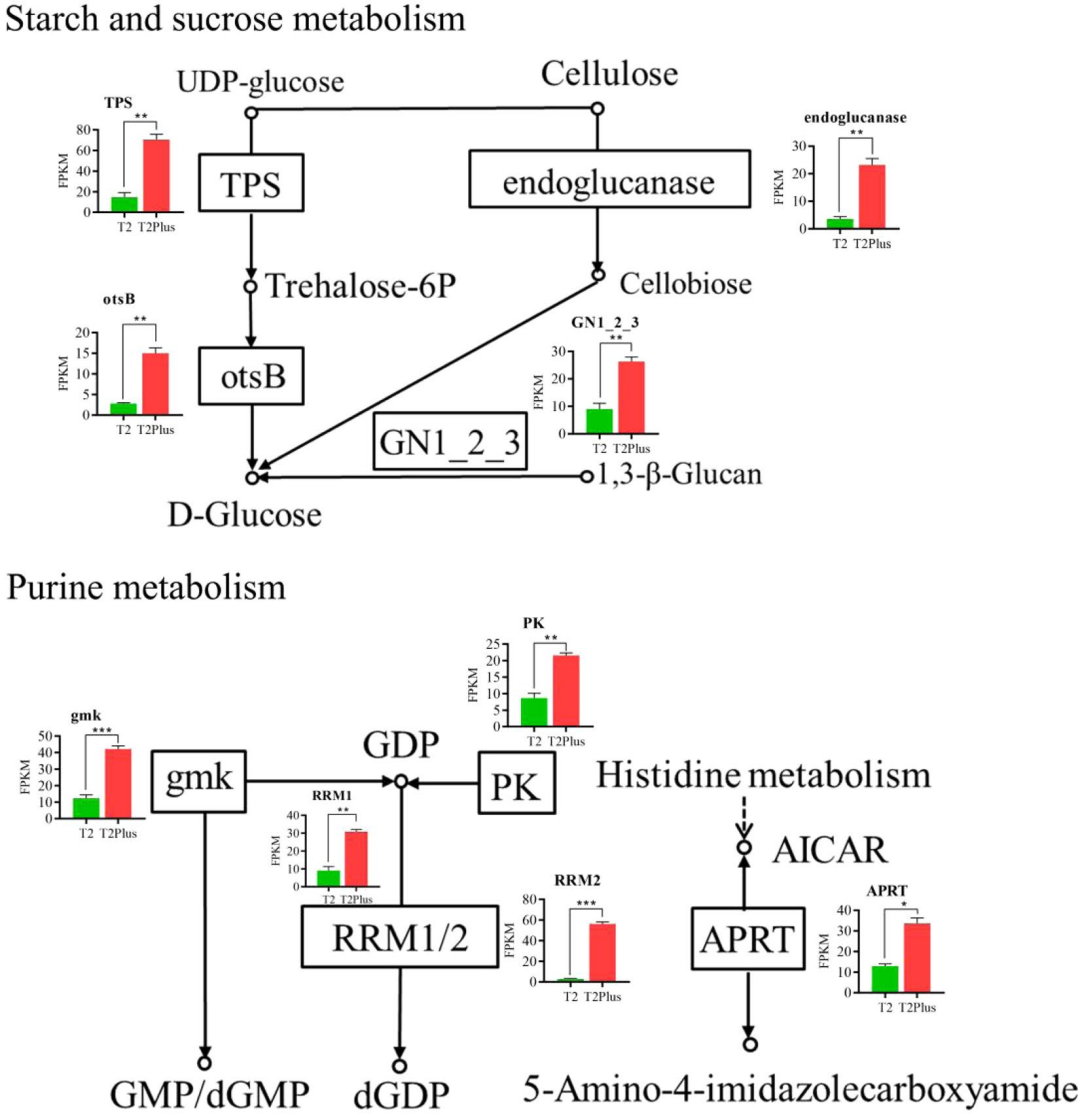
Expression profiles of DEGs related to ‘Starch and sucrose metabolism’ (top) and ‘Purine metabolism sugar metabolism’ (bottom) in T2 line and T2Plus line. All data shown reflect the average mean of three biological replicates (n = 3). Means with different letters in each treatment represent a significant difference of *P* ≤ 0.05. *p-value ≤ 0.05, **p-value ≤ 0.01, ***p-value ≤ 0.001.

The content of polysaccharides and guanosine, adenosine was higher significantly in T2Plus line than T2 line (Fig. 1d) and ‘Starch and sucrose metabolism’ and ‘Purine metabolism’ were enriched significantly by KEGG enrichment analysis. In ‘Starch and sucrose metabolism’ pathway, the expression level of genes TRINITY_DN19188_c0_g1 (trehalose 6-phosphate phosphatase, otsB), TRINITY_DN408_c0_g1 (glucan endo-1,3-beta-glucosidase 1/2/3, GN1_2_3), TRINITY_DN7111_c0_g1 (trehalose 6-phosphate synthase/phosphatase, TPS) and TRINITY_DN7933_c1_g1 (endoglucanase) in T2Plus line were higher significantly than T2 line (Fig. 4: top). The expression levels of these four DEGs were consistent with the trends of polysaccharides content, indicating that these four DEGs might play important role in promoting biosynthesis of polysaccharides. In ‘Purine metabolism’ pathway, compared with T2 line, the expression of genes TRINITY_DN18222_c0_g1/TRINITY_DN6388_c0_g1 (ribonucleoside-diphosphate reductase subunit M1/2, RRM1/2), TRINITY_DN3492_c1_g2 (guanylate kinase, gmk), TRINITY_DN5231_c1_g2 (adenine phosphoribosyltransferase, APRT) and TRINITY_DN972_c0_g1 (pyruvate kinase, PK) was increased significantly (Fig. 4: bottom). The result suggested that these five genes may play an important role in the bio-synthesis of guanosine and adenosine.

### Photosynthetic efficiency analysis

**Figure 5:**
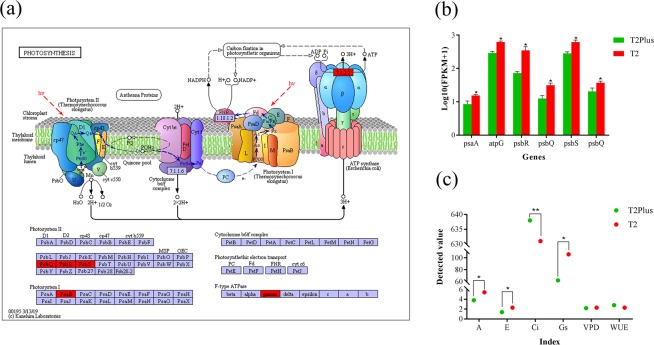
Photosynthetic efficiency analysis. (**a**) Six up-regulated DEGs in T2Plus of photosynthesis (red frame). (**a**) was obtained by mapping the DEGs into KEGG database (www.kegg.jp/kegg/kegg1.html) using the pathway mapping tool (https://www.genome.jp/kegg/tool/map_pathway2.html)^66^. (**b**) The expression levels of six up-regulated DEGs. (**c**) Parameters of the photosynthetic efficiency are measured. A: umol▪m^−2^▪s^−1^, E: mmol▪m^−2^▪s^−1^, Ci: ppm, Gs: mmol▪m^−2^▪s^−1^, VPD: mb, WUE: %. *p-value ≤ 0.05, **p-value ≤ 0.01. (**b**,**c**) were drawn by using the Origin 8.0 (Origin lab corporation, Wellesley Hills, Wellesley, MA, USA). The entire Fig. 5 was obtained by integrating the single (**a**–**c**) into a big figure using Adobe Illustrator CS6 software.

Photosynthesis is closely related to plant growth and development. The chlorophyll catabolic process and photosynthetic electron transport chain were enriched significantly in GO term enrichment analysis (Fig. 3c). In addition, a total of six DEGs associated with ‘Photosynthesis’ in KEGG pathway enrichment analysis were down regulated in T2Plus line (Fig. 5a,b). The results indicate that photosynthetic efficiency in T2Plus line were lower than that in T2 line.

In order to confirm this hypothesis, an analysis of photosynthetic efficiency was performed. A useful measure index of the photosynthetic efficiency of plants is ‘net assimilation rate’ (A), which is the rate of increase of dry weight per unit of leaf area^23^. The transpiration rate (E) related to photosynthesis reflects the ability of plants to absorb and transport, which was regulated by stomata size. Carbon dioxide (CO_2_) and water vapor are essential substances for photosynthesis, and the rate at which they enter stomata is measured by ‘Stomatal conductance’ (Gs)^24^. The ‘Concentration of CO_2_ intercellular’ (Ci) value was maintained at a relatively stable level through both Gs and the efficiency of the mesophyll cell to utilize the substrate CO_2_ and a lower ‘Ci’ value was indicated the better photosynthetic efficiency^25^. In high ‘Vapor Pressure Deficit’ (VPD) environment, reduction of ‘E’ would increase ‘Water Use Efficiency’ (WUE)^26^. Therefore, these six parameters (‘A’, ‘E’, ‘Gs’, ‘Ci’, ‘VPD’ and ‘WUE’) were measured. Compared with T2 line, the values of ‘A’, ‘E’ and ‘Gs’ were lower and ‘Ci’ was significantly higher in T2Plus line than that in T2line, (Fig. 5b; Table 2), suggesting that the photosynthetic efficiency of the T2Plus line is significantly lower than that of the T2 line.

**Table 2:** Photosynthetic efficiency analysis.

Line	A/umol▪m^−2^▪s^−1^	E/mmol▪m^−2^▪s^−1^	Ci/ppm	Gs/mmol▪m^−2^▪s^−1^	VPD/mb	WUE /%
T2Plus	3.8	1.4	638	62	2.2	2.8
T2	5.4	2.3	631	106	2.3	2.3

### Identification of DEGs associated with transcription factors

**Figure 6:**
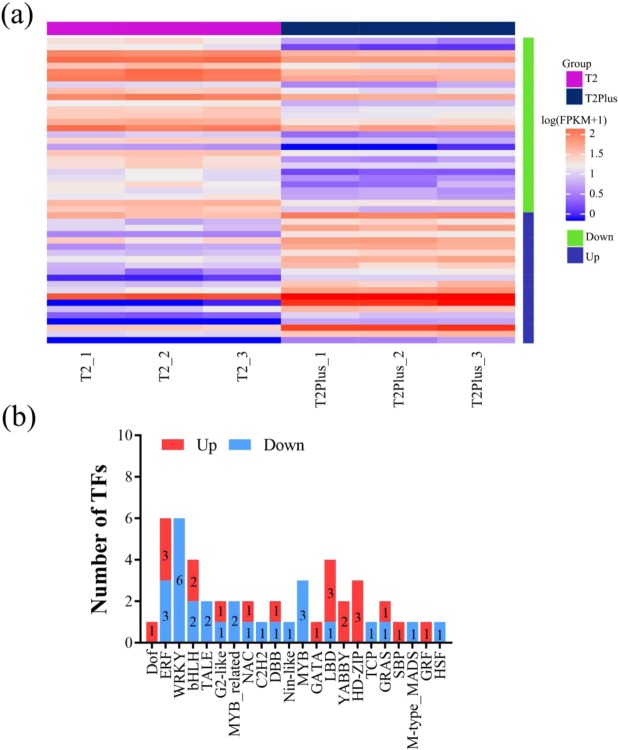
Identification and expression profile of differential expression TFs. (**a**) Expression profiles of differentially expressed TFs between T2 line and T2Plus line. (**b**) Distribution of differentially expressed TF families in T2Plus line. Red represents up-regulation of differential expression TFs. Blue represents down-regulation of differential expression TFs.

Transcription factors (TFs), as an essential parts of the organism, regulate almost each aspect of the organism’s life metabolism^27^. A total of 539 potential TFs were detected in the transcriptomes of *Pinellia* using Plant Transcription Factor Database (http://planttfdb.cbi.pku.edu.cn/prediction.php). To better understand the molecular mechanism of differences in transcriptome and characteristics between T2Plus line and T2 line, the differential expression of TFs were measured and a total of 49 genes (up: 21, down: 28) encoding TFs (including 22 TF families) with significant differences were obtained (Fig. 6). The significantly up-regulated TFs included three genes encoding ERF (TRINITY_DN21800_c0_g1, TRINITY_DN86071_c0_g1, TRINITY_DN98068_c0_g1), two genes encoding basic-helix-1oop-helix (bHLH) (TRINITY_DN3051_c0_g1, TRINITY_DN701_c1_g1), three genes encoding HD-ZIP (TRINITY_DN31663_c2_g1, TRINITY_DN439_c0_g2, TRINITY_DN4552_c2_g1). In addition, bHLH (TRINITY_DN1351_c1_g1, TRINITY_DN1628_c0_g1), ERF (TRINITY_DN11471_c0_g2, TRINITY_DN151_c1_g1, TRINITY_DN594_c1_g1), MYB (TRINITY_DN2319_c0_g1, TRINITY_DN2319_c0_g2, TRINITY_DN567_c0_g2), MYB related (TRINITY_DN14570_c0_g2, TRINITY_DN3364_c2_g1), WRKY (TRINITY_DN12750_c0_g2, TRINITY_DN3466_c0_g1, TRINITY_DN4327_c3_g1, TRINITY_DN45076_c0_g1, TRINITY_DN6277_c0_g1, TRINITY_DN6636_c0_g1) were belonging to down regulated TFs. These TFs might play significant roles in differences of transcriptome and characteristics between T2Plus line and T2 line. The basic information for researching the role of TFs in the promoting effect of biosynthesis of medicinal ingredients in *Pinellia* was provided by these findings.

## Discussion

*Pinellia*, as a traditional Chinese medicine, has a variety of pharmacological activities and a wide range of clinical applications. Due to the fact that the yield and quality of the *Pinellia* in different regions are different, the superior individual T2 line was obtained by screening cultivar from different regions. Next, we used tissue culture technology to detoxify T2 line, which increased the yield and quality of *Pinellia*. The chromosome-doubling technique, with colchicine-induced T2 suspension culture cells, was performed to generate polyploid *Pinellia* (sixteen ploid) T2Plus line (2n = 16 * 13 = 208) from octoploid T2 line (2n = 8 * 13 = 104). In T2Pus line, the phenotypes were significantly better, the length of the petiole was shorter, the diameter of the petiole was larger, the proliferation coefficient was lower and there was less biomass accumulation than with the T2 line. In addition, compared with T2 line, the contents of various medicinal components in the T2Plus line were increased, including polysaccharides, guanosine, adenosine, and ephedrine, which increased by 18.69%, 72.88%, 82.01% and 15.86%, respectively. These results suggested that doubling the chromosome of the Pinellia T2 line can noticeably increase its medicinal value.

The molecular mechanisms underlying the bio-synthesis of medicinal ingredients are poorly understood in *Pinellia*. The doubling chromosome of the T2 line inevitably leads to differences in the transcriptome. To investigate the relationship between the differential transcriptome and the bio-synthesis of medicinal components, we performed RNA-seq experiments using total RNA isolated from these two lines. A total of 1,656 up-regulated DEGs and 1,583 down-regulated DEGs (FDR ≤ 0.05, |log2FC | ≥ 1) were subjected to GO terms and KEGG pathways enrichment analysis.

In GO terms enrichment analysis, the DEGs in T2Plus line were predominantly involved in the cell-related terms, such as cell cycle, cell wall organization or bio-synthesis, cell division and mitotic cell cycle, which plays an important role in plant proliferation, growth and development. The phenomenon that the phenotype of T2Plus line were better than those of T2 line, indicating the cause due to the genes associated with these cell-related terms.

In KEGG pathways enrichment analysis of DEGs, a total of 18 metabolic pathways were significantly enriched (*P* ≤ 0.05). In these pathways, ‘Cysteine and methionine metabolism’, ‘Glyoxylate and dicarboxylate metabolism’, ‘Alanine, aspartate and glutamate metabolism’ belong to the basic metabolic pathway, which can provide precursors for the development and the synthesis of medicinal ingredients in *Pinellia*. There were 9 DEGs involved in ‘Ascorbate and aldarate metabolism’. Ascorbate is an antioxidant substance widely present in plant tissues and acts on plant cell resistance to oxidative stress, cell division and elongation^28^. Ascorbate is functioned an important role in plant metabolism and stress tolerance^29^. Interestingly, GO terms, photosynthesis, chlorophyll catabolic process and photosynthetic electron transport chain were associated with DEGs and the expression level of six DEGs related to ‘Photosynthesis’ pathway was significantly lower in T2Plus line than that in T2 line. In addition, compared with T2 line, photosynthetic efficiency was declined significantly in T2Plus line, suggesting that chromosome doubling reduces photosynthetic efficiency in T2Plus line.

In a cell or organism, many of the biological processes are influenced or controlled by the expression levels of genes regulated by transcription factors (TFs)^30^. In the present study, 539 potential TFs (21 up-regulated TFs and 28 down-regulated TFs) were detected in all of the transcriptome data. Among these TF families, MYB, bHLH, WRKY, LBD, HD-ZIP and ERF, accounted for a relatively large proportion, suggesting that these six TFs may play an important role in the transcriptome differences between T2 line and T2Plus line. MYB transcription factor family as one of the most abundant and diverse TF families in plant is widely involved in the regulation of different life processes, including cell cycle control^31,32^, secondary metabolism^33,34^, stress response^35–37^, etc. In current studies, bHLH transcription factors are involved in the regulation of various signal transduction and bio-synthesis and metabolism pathways, such as light signal transduction, hormone synthesis^38,39^, glandular hairs and root hair development and stress response^40,41^. WRKY transcription factors can regulate multiple biological processes by various networks regulation in plant^42^. For example, OsWRKY62 functions is both a negative regulator of innate immunity and a critical mediator of defense responses in *Oryza sativa* L^43^. Brassinosteroids (BRs) not only promotes the growth but also implicated in plant responses to drought stress. BR-mediated gene expression was promoted and drought responsive genes were inhibited by WRKY transcription factor families (WRKY46, WRKY54, and WRKY70) in *Arabidopsis thaliana*^44^. LBD transcription factor (lateral organ boundaries domain), a specific transcription factor^45^, play a key role in multiple processes of plant growth, development and metabolism in *Oryza sativa* L^46^, *Arabidopsis thaliana*^47,48^, *Vitis vinifera*^49^, *Citrus*^10^, *Sugar beet*^50,51^. HD-Zip transcription factor is composed of homeodomain (HD) and leucine zipper (LZ) and plays an important role in multiple aspects in *Arabidopsis thaliana*, such as development of leaf^52^, response to abiotic stress^53^ and regulation of seed development^54^. As a transcription factor widely present in animals and plants, ERF (Ethylene-responsive factor) has the following functions: regulation of plant growth and development^55^, participation in abiotic stress response^56–58^, and participation in biotic stress response^59,60^. These results suggest that these transcription factors may play an important role in the observed differences of phenotype and medicinal ingredients between T2 line and T2Plus line, and may increase the ability of resistance to biotic and abiotic stresses in T2Plus line.

## Materials and Methods

### Assessment of morphological characteristics

The plant materials used in this study were selected and used to breed a single plant with excellent and even quality, including octoploid *P. ternata* no.2 (T2 line) of peach leaves, from Heze Shandong province, China, and a new sixteen ploid *P. ternata* (T2Plus line) was obtained by artificial chromosome doubling using colchicine.

For analyzing phenotypic differences between T2 line and T2Plus line, the growth situation of plantlets growing in the field were recorded during a three-month period. The lengths and widths of leaves, petiole and plant height of 30 plantlets were measured. The fresh weights and dry weights and biomass of 6 plantlets were measured, and the length/width ratios and proliferation coefficients and dry matter rates were determined.

### Determination of medicinal ingredients

To investigate whether there are differences in the medicinal components of *P. ternata* in these two lines, the contents of proteins, polysaccharides, alkaloids and nucleosides in tubers of *P. ternata* were determined. The content of polysaccharide was measured by the optimized phenolic phenol method, protein content was obtained by ultraviolet spectrophotometry, and guanosine, adenosine and ephedrine in *P. ternata* were measured by high performance liquid chromatography (HPLC).

### Generation of *P. ternata* without contamination viruses or bacteria

Firstly, *P. ternata* was collected from outdoor and the petioles of *P. ternata* cleaned with tap water for 30 min. Then, the petioles of *P. ternata* were put into ultra-clean worktable and sterilized for 30 min by ultraviolet. These were immersed in 75% (v/v) ethanol for 30 s and a 0.1% mercury chloride for 5 to 10 min by means of intermittent agitation. Finally, the both ends of petioles were removed and the remaining petioles cut into 1 to 1.5 cm. The experiment bottles were filled up with 50 mL of autoclaved solid basal MS medium supplemented with 0.05 mg/L of NAA (α-Naphthaleneacetic acid) and 1.0 mg/L of 6BA (6benzyladenine) and 30 g/L sucrose and 6.0 g/L agar (Shanghai Yuhan Bio-tech Co. Ltd, China) at the pH of 5.8–6.2. Thereby, the aseptic tissue culture seedlings of *P. ternata* could be obtained.

### Photosynthetic efficiency

To investigate the effect of plant polyploidy on photosynthesis, a photosynthetic assay was determined with a portable photosynthetic analyzer (Ciras-3) with setting of the appropriate parameters. Leaves of the T2 line and T2 Plus line of *P. ternata* cultured at the same conditions were collected. The photosynthetic parameters were measured by placing the leaves on the Ciras-3 photosynthetic analyzer, and the experimental data read and recorded. The determination time was 9:30 a.m. on April 4, 2019. The measured parameters were as follows: (A), (E), (Gs), (Ci), (VPD), (WUE). Net Assimilation Rate (A, umol▪m^−2^▪s^−1^), Transpiration Rate (E, mmol▪m^−2^▪s^−1^), Internal CO_2_ (Ci, ppm), Stomatal Conductance (Gs, mmol▪m^−2^▪^−1^), Vapour Pressure Deficit (VPD, mb), Water Use Efficiency (WUE, %).

### RNA isolation, library construction, and sequencing

For RNA analysis, the tissue samples were collected from cluster plantlets of *P. ternata*. These two groups included the T2 line and T2Plus line, with three biological replicates, and T2 line was the control group. At 30 days after planting, seedlings are collected and frozen in liquid nitrogen for more than 3 min immediately and stored at −80 °C until use. Five seedlings collected from each group were mixed and thoroughly ground to powder with liquid nitrogen, and approximately 50 mg per pooled sample was prepared to use. Total RNA was isolated from each replicated sample according to the instructions of OmniPlant RNA Kit (CWBIO, Beijing, China). Purified RNA samples were quantified and qualified by the Nanodrop 2000 c Spectrophotometer (Thermo Fisher Scientific, USA) and agarose gel electrophoresis analyses, respectively. For each sample, an approximately 2 μg total RNA was used for the quality evaluation. Quantified using Qubit 2.0 Q32866 (Life Technologies, USA) was used to prepare the cDNA library.

We constructed the cDNA library according to the instructions of NEBNext Ultra RNA Library Prep Kit for Illumina (NEB, USA); brief steps are described below. Firstly, the mRNA was supplemented through oligo (dT) bind magnetic beads along with split into small parts., These were reserved as an template for the initial strand and subsequent strand cDNA synthesis. Then, the residual overhangs were transformed into the blunt ends through ligase and polymerase. The resulting peices were end revamped by introducing an “A” base to the 3′ ends of the cDNA. And the NEBNext connecters with a hairpin circle assembly were ligated to the fragments. To select suitable cDNA fragments, Agencourt AMPure XP 60 ml Kit (Beckman Coulter, USA) was applied to purify the library fragments. Thereafter, the products were augmented by polymerase chain reaction (PCR) for the preparation of sequence libraries. The 350–450 bp fragments were recovered using QIAquick Gel Extraction Kit (QIAGEN, Germany). It’s crucial of quality testing for the library to ensure that the library meets the requirements of sequencing. The built libraries were then sequenced via an Illumina HiSeqTM2500 platform.

### *De novo* assembly and functional annotation

We removed adaptor sequences, poly-N reads and low quality reads using FastQC (http://www.bioinformatics.babraham.ac.uk/projects/fastqc/) and Trimmomatic (V 0.36) software^61^ to obtain clean reads from raw data (containing more than 50% bases with Q ≤ 30). We used Trinity software to perform *de novo* assembly with a k-mer length of 25 and other default parameters^62^. The transcripts were clustered with a TGI clustering tool, and the longest transcripts were defined as unigenes.

We used BLASTX program with E-value of 10^−5^ to search functional annotation in the multiple protein databases, such as Swiss-Prot database^63^, Protein family (Pfam) database^64^, EggNOG database^65^, KEGG database^66^ and GO database^67^.

### mRNA abundance distributions

To obtain the average **F**ragments Per Kilobase of transcript per Million fragments mapped (FPKM), the abundances of all detected mRNAs of all non-zero samples were averaged. The average FPKM was the transformed by log2 and the R function density() with default settings was performed to estimate a kernel density of this log2 FPKM distribution. The R function (plot(), lines() and legend()) was performed to plot distributions of these kernel densities.

### Examination of differentially expressed unigenes

To calculate the abundance of unigenes, the clean reads sequenced from each sample were mapped back to the unigene library. To quantify the level of gene expression, RSEM was used to calculate FPKM of individual sample. Then, the DESeq R software package^66^ was used for comparing differential expression analysis between T2 line and T2Plus line. In multiple tests and analysis, we used FDR to identify the *P*-value threshold. Only genes with FDR ≤ 0.05 and more than two-fold change in expression between samples were considered as DEGs. On the basis of hyper-geometric test, GO enrichment analysis of DEGs was carried out by the topGO R package^68^. In addition, KOBAS software^69^ was applied to test the enriched pathway of DEGs. GO terms and KEGG pathways with corrected *P*-value ≤ 0.05 were recognized as obvious over-expression.

### Identification of transcription factors

Blastx was performed to search all of transcription factors from transcriptome using a plant transcription factor database (PlnTFDB^70^; http://plntfdb.bio.uni-potsdam.de/v3. 0/downloads.php) with a cut-off *E*-value of 1.0 e^−10^ to identify TFs^71^.

The all datas have been uploaded to the GEO database and the accession number was GSE140998.

## Supplementary information

supplementary information.

Dataset S2.

Dataset S3.

Dataset S4.

Dataset S1.
