# Supplementary material for: Phenotypic and Transcriptomic Analysis of Two Pinellia ternata Varieties T2 line and T2Plus line
Source: Sci Rep. 2020 Mar 12;10:4614. doi: 10.1038/s41598-020-61512-2 (PMC7067869; doi:10.1038/s41598-020-61512-2)
Supplement: Supplementary file 1 — supplementary information. [file 41598_2020_61512_MOESM1_ESM.pdf]

Phenotypic and Transcriptomic Analysis of Two *Pinellia ternata* Varieties T2 line and T2Plus line

Jun Lu[1, 3], Jian Ning Liu[2], Surendra Sarsaiya[3], Gregory Joseph Duns[1,4], Jing Han[1], Leilei Jin[1], Jishuang Chen[1, 3, *]

[1]*College of Biotechnology and Pharmaceutical Engineering, Nanjing Tech University, Nanjing 211800, Jiangsu, China*

[2]*KeGene Science & Technology Co. Ltd., Nantianmen Middle Road, Tai'an 271018, China*

[3]*Bioresource Institute for Healthy Utilization, Zunyi Medical University, Zunyi 563000, Guizhou, China*

[4]*AirChem Consulting and Research, London Ontario, N5X OE2, Canada*

*Corresponding Author: Jishuang Chen, Email: biochenjs@njtech.edu.cn

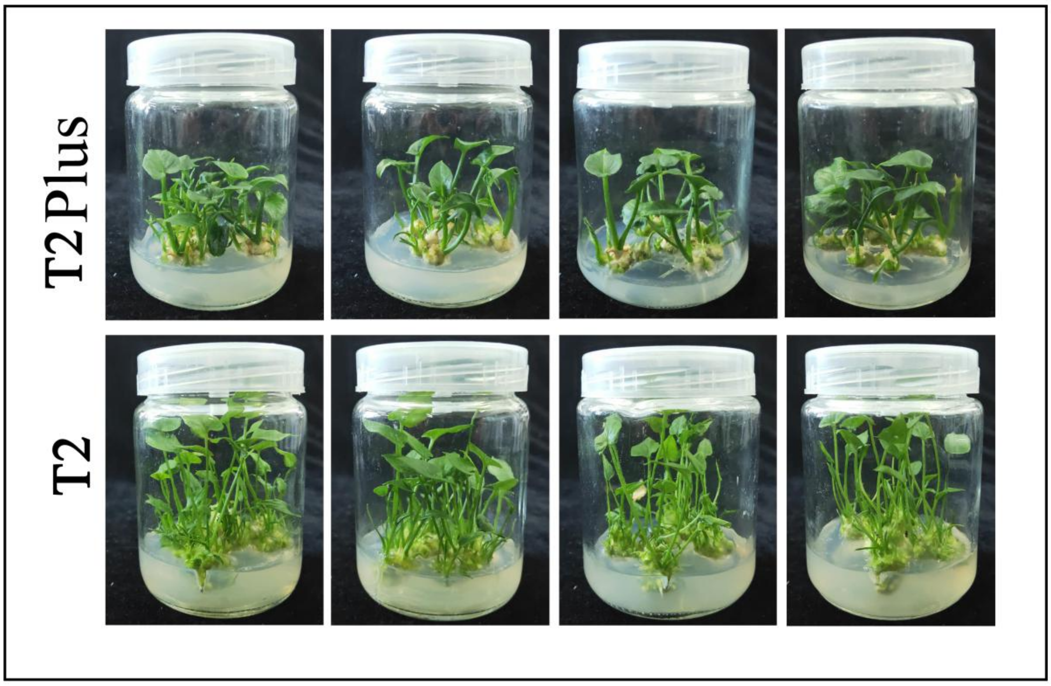

Supplementary Figure 1 | The growth state of T2 and T2Plus lines at the leaf proliferative stage.

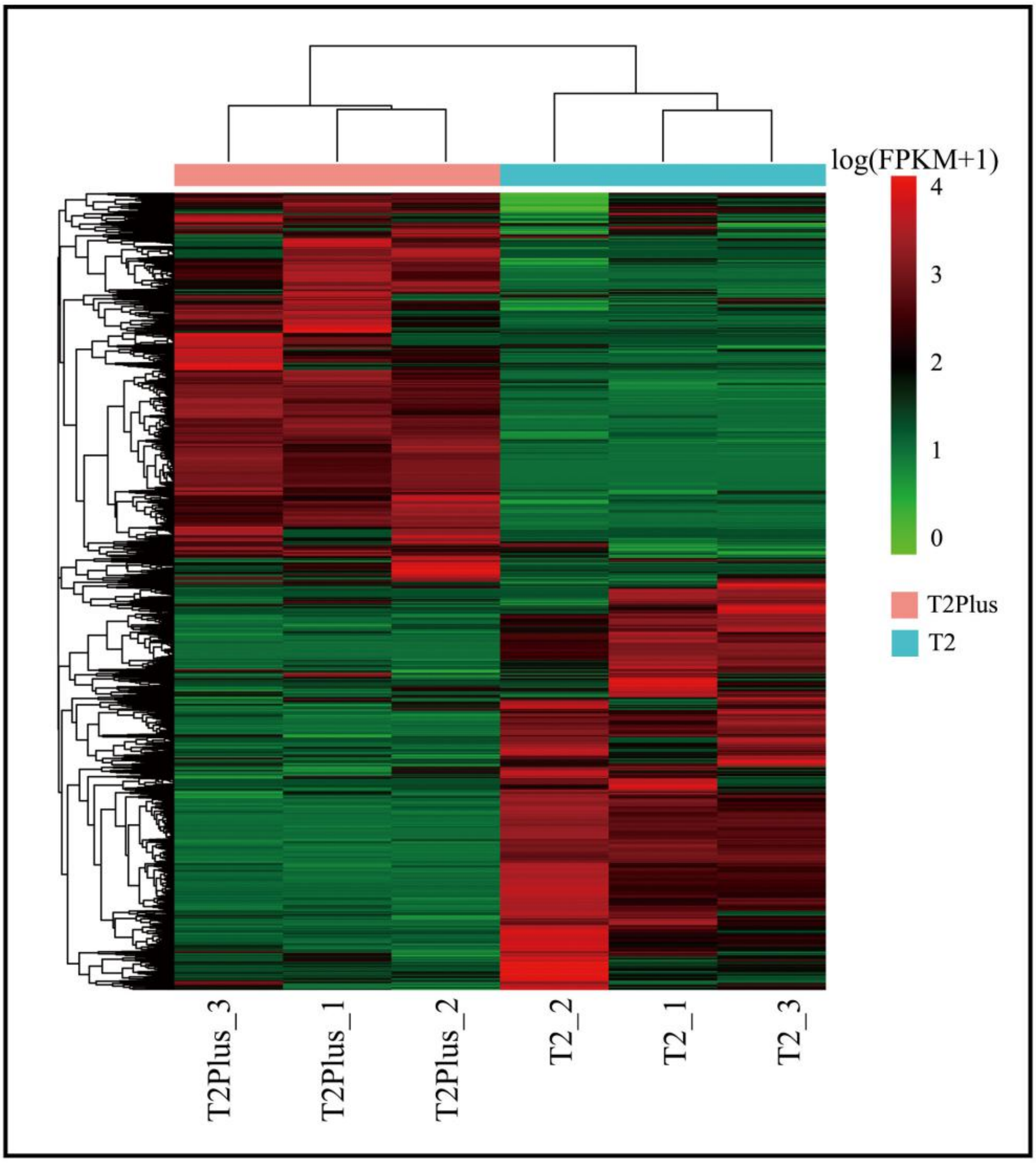

Supplementary Figure 2 | Heatmap for expression level of all unigene in T2 line and T2Plus line. FPKM values increase gradually according to the order of green, black and red.
